# The Interest Profiles and Interest Congruence of Male and Female Students in STEM and Non-STEM Fields

**DOI:** 10.3389/fpsyg.2019.00897

**Published:** 2019-04-30

**Authors:** Bernhard Ertl, Florian G. Hartmann

**Affiliations:** Department for Education, Universität der Bundeswehr München, Neubiberg, Germany

**Keywords:** STEM students, non-STEM students, vocational interests, RIASEC, choice of major, congruence, O^∗^NET, large scale study

## Abstract

The goal of the following study is to investigate whether first-year students in STEM fields that have a low proportion of females (STEM-L) show vocational interests that fit their vocational aspirations. To place our investigation into a broader context, we compared students in STEM-L with students of STEM subjects with a medium proportion of women (STEM-M) as well as with other subjects with a medium or a high proportion of females. We analyzed their vocational interests, vocational aspirations and their interest congruence. In both the comparison regarding interest profiles and the comparison of vocational aspirations, we focused on the things-orientation and people-orientation, all while taking respective gender differences into account. Following the suggestion from previous studies, in a further step we differentiated between subjects within STEM-L. Using data from the German National Educational Panel Study (NEPS), we analyzed the interest congruence of 5,530 male and 7,406 female students in STEM majors (with a low or medium proportion of women) and non-STEM majors (with a medium or high proportion of women). Students from different subjects showed different magnitudes regarding their things- and people-orientation. STEM-L students had a high things-orientation and a low people-orientation regarding both their interests and aspired occupations. Students of STEM-L and STEM-M showed a lower interest congruence than students from other subjects. With the exception of education, gender differences regarding the people- and things-orientation also existed within most of the subjects. Gender differences partly remain when distinguishing between the different subjects within STEM-L. And so, the result that not all STEM-L subjects are “created equal” is discussed in the context of their theoretical and methodological aspects.

## Introduction: Contexts of Females in Stem

Science and technology are drivers of societal benefit. This is one of the reasons why the EU Commission established the goal of increasing the number of STEM (**s**cience, **t**echnology, **e**ngineering, and **m**athematics) graduates (Hingel et al., 2008, p. 10). However, the proportion of students enrolled in STEM subjects hasn’t noticeably changed within the past decade (Eurostat, 2018), and the proportion of females in STEM remains low (Eurostat, 2018). This phenomenon requires a deeper look into the STEM subjects and the students (both female and male) who take them to potentially identify any peculiarities that may help achieve measures to encourage more students and especially more females to pursue a career in STEM.

When first observing the notion of STEM, it’s important to acknowledge that this term is somewhat blurry with respect to its subjects. Although the EU Commission (Hingel et al., 2008) only focuses on (physical) science, technology (including engineering), and mathematics, newer publications (European Commission, 2015) also include life sciences such as medicine (also Eccles and Wang, 2016). Publications from the United States (e.g., Su and Rounds, 2015) furthermore include social sciences. These gray areas provide challenges when analyzing gendered pathways into STEM. Engineering subjects have low proportions of females, often far less than 30% (see Su and Rounds, 2015; Destatis [Statistisches Bundesamt], 2018b). In contrast, social sciences like education have very high proportions of females, ranging in some cases above 70% (ibid.). So, any analysis that focuses on females in STEM needs to acknowledge that there is variance even within the STEM fields themselves.

Recent research has often clustered STEM subjects according to different criteria to deal with this heterogeneity and achieve more differentiated insights into the characteristics of STEM students (e.g., Ertl et al., 2014, 2017; Eccles and Wang, 2016; Watt et al., 2017). For example, Eccles and Wang (2016) grouped health, biological, and medical sciences, contrasting them with mathematics, physics, engineering, and computer sciences. They found that differences in subject choice for one of both groups resulted primarily from gender differences in occupational and lifestyle values. Ertl et al. (2014) distinguished STEM subjects with respect to their proportions of females, finding differences with respect to motivation, academic self-concept, and the impact of stereotypes. Watt et al. (2017) focused the subject groups of mathematics, physics, chemistry, and biology, revealing differences in gendered processes of influence by prior mathematical performance, motivation, and mothers’ perceptions. Recent research in sum has found differences in the student characteristics between the different STEM subjects or subject groupings.

Su and Rounds (2015) followed an approach of classifying STEM subjects very narrowly according to an interest profile that distinguishes the dimensions of *things* and *people*. Based on this profile, they projected a proportion of females for different STEM subjects and compared this with their actual proportion. This projection fit quite well in several cases, even though it showed a noticeable deviation in the fields of engineering and computer sciences in which it overestimated the proportion of women. This was also the case in applied mathematics and medical services where it underestimated the proportion of females (Su and Rounds, 2015, p. 15). Thus, they were able to show on a macro/subject level that the interest profile of a subject corresponds to the proportion of females within it.

This paper examines the vocational interests of individuals on a micro level and aims at revealing characteristics of interest profiles and their fit to aspired occupations. It will classify students’ vocational interests according to the RIASEC model of Holland (1997) and compare these profiles with the RIASEC profiles of the respective students’ vocational aspiration. The paper will apply a vector-based measure of congruence according to Eder (1998) that considers the Euclidean distance of the two interest vectors as congruence measure (see Prediger, 1982; Tracey and Sodano, 2013). This approach will reveal how far the individual has an interest congruence with his or her vocational aspiration, providing a more individual perspective than the comparison of subject-level interest profiles and proportions of females.

This investigation acknowledges the reported differences of students in STEM subjects. These may be a result of the proportion of females within a subject and/or its broader field. The paper will first classify subjects at the finest possible level according to their proportion of females: low (with a proportion of females less than 30%), medium (with a proportion of females between 30 and 70%), and high (with a proportion of females higher than 70%). It will then distinguish the broader subject area. The focus of this paper is on the STEM subjects of physical sciences, technology, engineering, and mathematics. However, for a point of comparison, to compare the results with previous research, and to interpret the results better within a broader context, the paper will also include the subject groups of medicine, economics, education, and languages.

## Vocational Interests and Career Choices

Dealing with the issue of why so few females go into STEM means starting with models of why individuals decide on specific career paths. The following will briefly introduce three theoretical models that have different emphases on explaining these career paths and at the same time are well compatible with each other. We will first describe Holland’s (1997) theory of occupational choice that deals with an individual’s interests as the basis for selecting an appropriate (congruent) occupation. This model emphasizes the aspect of vocational interests as a crucial factor for career decisions. The Gottfredson (1981, 2005) model of circumscription and compromise highlights the developmental process behind the individual’s occupational choice. Her model introduces the issue of sex-type regarding different occupations that may shape an individual’s choice based on an evaluation of how far an occupation may be considered “typical” for males or females. Here, the model describes the exclusion of what are considered “inappropriate” occupations at an early developmental stage. As a third approach, we will cite the social cognitive career theory (SCCT; Lent et al., 1994; Lent, 2013; Lent and Brown, 2013) that considers the perspectives of person-environment fit approaches (e.g., Holland, 1997) as well as developmental career theories (e.g., Gottfredson, 1981, 2005) which additionally includes cognitive variables being less stable and therefore more malleable than personality dispositions (Lent, 2013; c.f. Hartmann, 2018).

### Types of Vocational Interests

Holland’s (1997) theory of occupational choice focuses on vocational interests and distinguishes six ideal types: Realistic (R), Investigative (I), Artistic (A), Social (S), Enterprising (E), and Conventional (C). These six types are not only used to describe an individual’s personality including vocational interests. They also characterize potential work environments. According to Holland (1997), people seek work environments that fit their vocational interests. This means that people who resemble the realistic (R) personality type are interested in mechanical or technical activities. They prefer working with tools or machines. Therefore, they are supposed to choose a realistic occupation that includes these tasks and objects. For example, given realistic interests, it would be a consistent occupational choice to become a surveyor or radiologist. The investigative (I) type are people interested in mathematical and scientific activities. They prefer occupations like aerospace engineer or general internist. Artistic (A) individuals are interested in creative and artistic activities. Occupations such as architectural drafter or geneticist are well suited to their type. Social (S) people are interested in activities that emphasize social interaction and interpersonal relations. They enjoy teaching or helping other people. Following their interests, they could become music therapists or midwives. Enterprising (E) persons are interested in leading and convincing other people. They prefer occupations like clinical research coordinators or natural sciences managers. People resembling the conventional (C) type follow their preference for ordering and repetitive tasks and choose occupations like actuary or electronic drafter. All these occupations can be assigned to the broadest context of STEM fields (O^∗^NET OnLine, 2018), making clear that STEM occupations and their corresponding training paths can be very different in terms of the required vocational interests (see also Su and Rounds, 2015). According to Holland (1997) students who have interests in STEM fields should choose STEM work environments as their courses of study, and later on as their occupations. The inverse is also true: STEM work environments should choose students or graduates who display STEM interests. Empirical evidence indicates that although investigative interests are crucial for going into STEM, very different interest profiles can cause people to choose STEM subjects or occupations; these are not all created equal, especially when it comes to the question of whether the work environments are typically male or female. In other words, STEM fields have gender differences both within specific subject-related interests as well as differences in the actual percentage of women. Both gender-related differences can be explained by the things-orientation (R) and the people-orientation (S) of the STEM fields (Prediger, 1982), with the things-orientation attracting more men and the people-orientation attracting more women. Consequently, female students tend to be interested in and choose STEM fields that are people-oriented and avoid STEM fields that are things-orientated (Su et al., 2009; Su and Rounds, 2015).

When it comes to the question of how vocational interests emerge, Holland (1997) describes a rather general model in which an individual’s career-related development is based on the interplay of genes and environmental influences.

### Development of Vocational Aspirations

Gottfredson (1981, 2005) looked deeper into the development of vocational aspirations, providing a specific explanation for the development of gender-specific differences regarding the choice of occupations. In her model of circumscription and compromise, an individual’s career choice is described as a process that is mainly based on two personal developmental processes: the individual’s cognitive growth, which is the development of cognitive skills, and the self-creation, which is the individual’s development of a self-concept. The development of the self-concept involves the gradual development of cognitive skills, as well as the successive exclusion of occupations that are are no longer compatible with the current self-concept. At an early developmental stage (orientation to sex roles; 6–8 years of age) children classify people as well as work environments as “male” or “female.” They become aware of their own gender and exclude occupations that are too male or too female and therefore outside of their tolerable sex*-*type boundary. Individuals then exclude occupations whose prestige is too low (tolerable-level boundary) and whose requirements are too high (tolerable-effort boundary). Gottfredson (2005) claims that the tolerable-level boundary is more important for men’s career choices because typically male occupations show a higher variance with regard to their prestige level than typical female occupations. All three boundaries together define the individual’s social space on the cognitive map of occupations within which occupations are eventually chosen based on personal interests in the sense of Holland (1997). If an individual is not able to find a work environment that is compatible with his or her specific self-concept, the restrictions are solved successively, with the tolerable sex-type boundary being perpetuated most strongly. Along with the mere congruence of occupational interests and occupational characteristics of the Holland (1997) model, Gottfredson (1981, 2005) understands gender as a category of occupational choice. This is a category that may restrict a woman’s social space, perhaps making the choice of a STEM field unlikely even if her interests do in fact match it. The empirical evidence for Gottfredson (1981, 2005) theory is equivocal with studies often lacking appropriate methods when investigating the processes of circumscription and compromise, especially when trying to assess individuals’ social space (Gottfredson, 2005; Junk and Armstrong, 2010).

With the objective of bringing more women into typically male occupations, the focus becomes which STEM occupations often are more “malleable,” and have attributes that influence occupational choice.

### Socio-Cognitive Career Theory

Here, the SCCT (Lent et al., 1994; Lent, 2013; Lent and Brown, 2013) provides a framework that is compatible with both person-environment fit approaches and developmental career theories. The SCCT is based on Bandura’s (1986) social cognitive theory. It highlights three cognitive variables that are less stable than personality variables and that are related to individuals’ interest development and career choices. These variables are self-efficacy beliefs, outcome expectations, and personal goals. Self-efficacy beliefs are personal assessments of one’s own potential for being able to perform certain actions and cause related outcomes. Outcome expectations are evaluations of the results that possibly come with certain actions; they can be influenced by self-efficacy beliefs. Personal goals refer to the actions a person wants to carry out or to the outcome a person wants to produce (Lent, 2013). The SCCT includes four models that focus on different aspects of vocational behavior. According to the *choice model*, the development of vocational interests depends on self-efficacy expectations and outcome expectations, which are themselves the result of an individual’s learning experiences. The learning experiences in turn depend on personal variables like gender or ethnicity and background influences like gender role socialization or the presence of different career role models. For example, if a person is encouraged to perform the realistic activities suggested by his or her parents and experiences positive outcomes, he or she may develop a high self-efficacy and positive outcome expectations toward realistic fields. He or she may develop strong realistic interests as a result. Vocational interests in this model have an impact on choice goals and actions (e.g., studying a STEM subject) with outcomes (e.g., exam results) that are the basis for new learning experiences. Along with the more distal background influences, there are environmental variables that can directly impact choice goals (and choice actions) or even moderate their relation to vocational interests (e.g., if a young woman is lacking emotional support for a career into STEM, the impact of her realistic orientation on her choice goals may become weaker). The choice model of the SCCT (Lent et al., 1994; Lent, 2013; Lent and Brown, 2013) could be confirmed by several empirical studies (Lent et al., 1994; Rottinghaus et al., 2003; Sheu et al., 2010). With regard to gender-specific differences, males show a higher level of confidence toward realistic and investigative issues (mechanical, outdoor/physical, mathematics, science) than females (Betz and Wolfe, 2005).

### Consequences for Investing Females’ Careers in STEM

Although the models described provide frameworks for career choice processes in general, more research is needed on the impacts of the factors on females’ choices for or against STEM careers. This research should start from the macro perspective of Su and Rounds (2015), with “men preferring working with things and women preferring working with people” (Su et al., 2009, p. 880). This also includes a closer look at the interests of the individuals within the different STEM fields. Furthermore, much of the previous research was built on convenience samples comprised of students from a specific university or region. This makes it essential to systematically analyze samples of male and female students that either go into STEM or something else. It’s also necessary to consider that not all STEM fields are created equal regarding vocational interests; this of course includes both the people-orientation as well as the things-orientation (Su and Rounds, 2015).

As it is, men are more interested in things (R) and women are more interested in people (S) (Su et al., 2009; Su and Rounds, 2015; Morris, 2016). Su and Rounds (2015) conclude – in line with Gottfredson (1981, 2005), Holland (1997), and the SCCT (Lent et al., 1994; Lent, 2013; Lent and Brown, 2013) – that interests are strong predictors of vocational choices. Women tend to choose social work environments and avoid STEM fields, especially those that only require realistic interests. Su and Rounds (2015) suspect two parallel processes lying behind the choice of social work environments and the avoidance of STEM fields by females. Both processes take into account quantitative skills mostly required to be successful in STEM. On the one hand, they assume a *constraining* process within which women may develop strong people-orientated interests and skills, but weak things-orientated interests and weak quantitative skills. Because these women have lower quantitative skills than others, they do not go into STEM fields or do not have the option to do so (c.f. Gottfredson, 1981, 2005). On the other hand, they assume a *broadening* process within which females who develop strong realistic interests and quantitative skills also have a better chance than males to develop social interests and people-orientated skills, reducing the chance to go into STEM fields (Wang et al., 2013; Woodcock et al., 2013).

## Measuring the Congruence Between a Person’s Interests and the Vocational Aspiration

The theory of Holland (1997) postulates that a person seeks the best fit between his or her interest profile and the profile of his or her (aspired) environment. Testing this hypothesis means creating profiles of both persons and environments and calculating the fit between both (this is also called *congruence*). There are several methods available to do this.

### Representing Interest Profiles and Profiles of Work Environments

**FIGURE 1:**
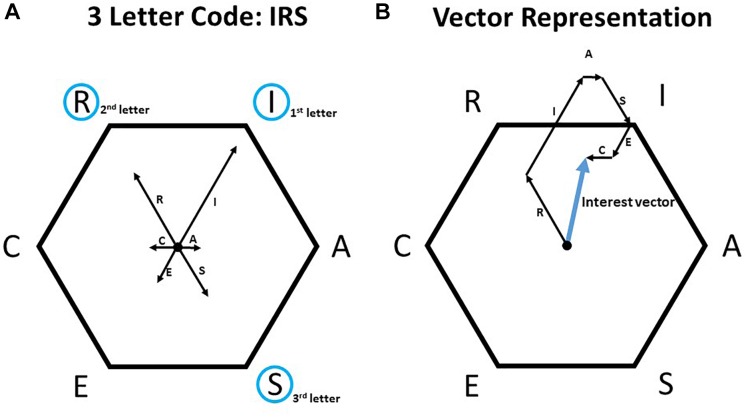
Representation of the interest profiles using the three-letter code (**A**; Ertl and Hartmann, 2019, reprinted with permission) and using an interest vector **(B)**. The values in the single RIASEC dimensions are represented by the thin arrows, while the interest profile for the three-letter code is visualized by the blue circles and the numbers indicating the position of each letter **(A)**. This is represented by the thick blue arrow for the vector representation **(B)**.

If inventoried interests (Super, 1957) in the sense of Holland (1997) are available, the complete interest profile usually consists of six scores, each indicating the similarity of a person to one of the six RIASEC types. Based on the six scores two different methods are commonly used to represent an interest profile. The first method uses the three dimensions the person is most similar to and creates a three-letter code (Holland, 1997). For example, if a person resembles the investigative (I) type the most followed by the realistic (R) type and the social (S) type, the person is assigned the code IRS (see Figure 1A). The second commonly used method relies on Holland’s (1997) assumption that the RIASEC types can be mapped onto a regular hexagon, describing each interest value as a single vector directed toward the respective corner in the hexagon (see small arrows in Figure 1B). The interest profile can then be described by a vector resulting from the sum of the six single vectors (see thick arrow in Figure 1B). This vector has a length that describes the differentiation of the profile, and a main direction toward one of the RIASEC dimensions (Eder, 1998; c.f. Prediger, 1982).^1^ Figure 1 illustrates the differences between interest profiles represented by a three-letter code (Figure 1A) and by an interest vector (Figure 1B). Representing interest profiles by the three-letter code (Figure 1A) allows a ranking of the three main dimensions (e.g., IRS). However, it does not address the magnitude of the differences between the dimensions, nor does it take into account the information given by the three remaining RIASEC dimensions. In contrast, the vector representation (Figure 1B) includes all the dimensions for representing an interest profile. Along with three-letter codes (e.g., IRS), an interest profile can be represented by the dominant type only (e.g., I) or by a two-letter code indicating the dominant and the second dominant type (e.g., IR). Depending on the data available, the methods of one-, two- and three-letter codes as well as the method of interest vectors can also be applied to work environments. The characterization of a work environment is mainly based on three different methods: expert ratings, assessing respondents’ personality, and assessing the content and demands of work environments. In the case of expert ratings, occupational analysts characterize work environments and derive an environmental profile (e.g., three-letter codes or numerical profiles) using occupational information and Holland’s (1997) RIASEC types (e.g., see Rounds et al., 2013). If the respondents’ personality is assessed, Holland’s (1997) environmental assessment technique (EAT) can be applied, i.e., the information about respondents having the same occupation is used to derive a one-, two-, or three-letter code that reflects the distribution of the personal dominant RIASEC types in that occupation. In these cases, the work environment is characterized by the personality of its inhabitants. A third option for creating an environmental profile is to ask inhabitants to fill out tests like the Position Classification Inventory (PCI; Gottfredson and Holland, 1991) that contains items assessing the content and demands of an occupation. The information from single questionnaires can be aggregated again to a one-, two-, or three-letter code. Given numerical profiles, an interest vector can also be calculated using the three methods described. An online source containing extensive information about work environments is available from O^∗^NET OnLine (2018).

### Analyzing Congruencies Between a Person and the Environment

**FIGURE 2:**
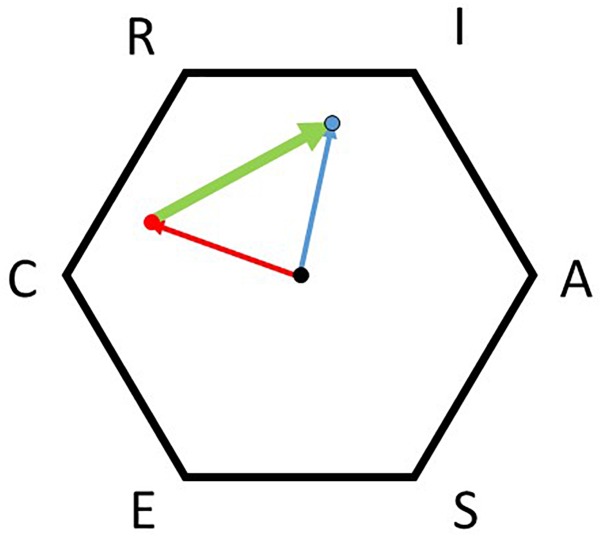
Representation of the congruence between a person and his or her environment. Blue represents the person’s interests, red the environment’s, and green the measure of congruence.

A wide selection of different congruence indices are available for analyzing the congruence between a person’s interest profile and the profile of his or her environment (e.g., first-letter agreement based on the hexagon by Holland, 1963; two-letter agreement index by Healy and Mourton, 1983; Z-S index by Zener and Schnuelle, 1976; M-Index by Iachan, 1984; ranked comparison congruence scale by Robbins et al., 1978). These algorithms based on Holland’s codes differ for example in the consideration of the hexagonal model; in the number of letters considered; and in the weighting of differences and letter positions (c.f. Tracey and Sodano, 2013; Hartmann, 2018). Studies comparing different congruence indices reveal that their similarities range from *r* = 0.05 to *r* = 0.98 (Camp and Chartrand, 1992; Brown and Gore, 1994; Young et al., 1998) causing different results concerning the relation of congruence with outcome variables like occupational satisfaction (e.g., Assouline and Meir, 1987; Tranberg et al., 1993; Young et al., 1998; Tsabari et al., 2005). More recent studies apply the profile correlation (Allen and Robbins, 2010; Tracey et al., 2012; Wille et al., 2014; Xu and Tracey, 2016), the angle difference of two vectors in the hexagon (angular displacement; Tracey and Robbins, 2006; Hartmann et al., 2015), or the Euclidian distance of two vectors in the hexagon (Tracey and Robbins, 2005, 2006; Tracey et al., 2005, 2012, 2014; Neumann et al., 2009; Wille et al., 2014) to measure congruence between two profiles using full profile information. Studies using more than one of those methods reveal a rather moderate similarity between them (e.g., Tracey and Robbins, 2006; Tracey et al., 2012; Wille et al., 2014; Hartmann, 2018). All three methods are related in expected ways to their outcomes (Tracey and Robbins, 2005; Tracey et al., 2005, 2012; Durr and Tracey, 2009; Neumann et al., 2009; Allen and Robbins, 2010). According to Tracey and Sodano (2013) the Euclidean distance is preferable because of its “ease of calculation, the unneeded assumption of independence of scales, and the easy extrapolation to more than two dimensions…” (p. 115). An application of the Euclidean distance is illustrated in Figure 2. The blue arrow represents the vector of the person’s interests with the three main dimensions of IRS, while the red arrow represents the environment’s profile with the main dimensions of CRS. The congruence between both profiles is estimated by the difference between both vectors (see the thick green arrow). With a higher congruence of person and environment, the difference and, consequently, the green arrow gets shorter until its value is zero, indicating maximum congruence. With a lower congruence, the green arrow grows until its length reaches twice the diameter of the hexagon for maximum divergence (see also Eder, 1998).

### Context for Investigating Interest Congruence for STEM Professions

STEM fields are of great importance to the future development of society. However, especially the number of females who choose to take STEM subjects or STEM professions is limited. One possible explanation for the low proportion of females is that the interest profiles of women do not match the profiles of STEM fields.

The Holland (1997) model describes a well-received background for assessing the fit between the individual and her or his vocational environment. Yet, while the model is well received in research and career counseling, very few is known about applying this model for explaining STEM careers. A Scopus inquiry for “vocational interest STEM” in March 2019 just resulted in 36 hits of which just five deal more specific with this issue. Most of these five discuss the aspects of differences in the things and people orientation (e.g., Su et al., 2009; Yang and Barth, 2015) while one of them (Babarović et al., 2018) maps specific interests for STEM into the RIASEC hexagon. Thus, it is essential to analyze the interest profiles of females and males in STEM and also to compare these with other occupational fields to get reliable knowledge about the characteristic interest profiles of females in STEM.

This is even more true when looking at the aspect of interest congruence. Among other topics, previous research dealing with interest congruence focused on the measurement of interest congruence (e.g., Camp and Chartrand, 1992; Brown and Gore, 1994), its connection to outcome variables (e.g., Tsabari et al., 2005; Nye et al., 2012) or the congruencies between the individual’s interests and the interests of her or his socialization group (e.g., Luttenberger et al., 2014; Etzel et al., 2018; Hartmann, 2018; Ertl and Hartmann, 2019). So far, research has barely dealt with the more specific topic of interest congruence within STEM fields: the Scopus research just revealed one relevant hit that investigates interest congruence as one variable among others for predicting IT job satisfaction (Carpenter et al., 2018). Thus, also in the area of interest congruence more research is needed that investigates how far the interest congruence of females and males varies within STEM and distinguishes from other fields.

## Methods, Modes of Inquiry, and Research Questions

### Research Questions

This leads to the following research questions and hypotheses:

1. To what extent do female and male students of selected STEM/non-STEM fields distinguish themselves with respect to their RIASEC interest profiles?
2. To what extent do female and male students of selected STEM/non-STEM fields distinguish themselves with respect to the congruence between their interests and their vocational aspirations?

### Research Rationale and Hypotheses

Previous research has shown that it is crucial to differentiate within STEM fields when characterizing students’ interest profiles. Therefore, using Holland’s (1997) RIASEC model, the research questions analyze to what extent female and male students from selected STEM and non-STEM subjects with different proportions of women differ with respect to their interest profiles, their vocational aspirations and the congruence between their interests and aspirations. The following will apply a vector-based analysis while considering the aspects of conceptualizing interests, profiles, and congruence (e.g., Eder, 1998).

The first research question is comprised of a descriptive part that characterizes the interests and vocational aspirations of different student populations. Its results will provide insights into vectors as well as into the predominant three-letter codes representing the respondents’ interests and vocational aspirations. In a second step it aims to analyze the extent of gender differences regarding interests and vocational aspirations. According to Su and Rounds (2015) one STEM field can be very heterogeneous in terms of its things-orientation and people-orientation and, in consequence, regarding the actual proportion of females. Here, we focus on the STEM field with a low proportion of females (STEM-L), comparing it with other fields of study. In addition, we look deeper into the gender differences within STEM-L by differentiating between subjects. In the context of the first research question we aim to test three hypotheses:

1. Students in STEM-L show higher realistic interests and lower social interests than students in other fields.
2. Students’ vocational aspirations in STEM-L show a stronger realistic orientation and weaker social orientation than students’ vocational aspirations in other fields.
3. Within STEM-L, female students show lower realistic and higher social interests than male students.

We generally assume a higher homogeneity within STEM-L, which means that interest differences decrease.

Research question 2 analyzes the match between the interests of the individuals and the interest profile of the vocational aspiration, which is the congruence between the individual and environment. Generally, we would hypothesize that people seek occupations that suit their interests (Gottfredson, 1981, 2005; Lent et al., 1994; Holland, 1997; Lent, 2013; Su and Rounds, 2015). Although there may be a higher chance for female students in STEM-L to have competing social interests that reduce their congruence, we assume that these females are able to seek a job that fits their interest profile. In the context of research question 2, we aim to investigate two alternative assumptions:

a. According to Su and Rounds (2015) we would assume that there are no differences regarding congruence between different fields of study.
b. Students have to overcome obstacles in subjects where they are under-represented. These may include stereotypes for females in STEM (see Ertl et al., 2017) or the low prestige of jobs for males in education and languages (see e.g., Gottfredson, 1981). According to SCCT (Lent et al., 1994) a higher congruence in interests could mitigate these obstacles to students choosing the respective field.

## Materials and Methods

Data sources used for the analysis were the cohort of first year students (SC5:10.0.0) of the German National Educational Panel Study (Blossfeld et al., 2011; see also acknowledgments) that started in the winter term of 2010/2011 (FDZ-LIfBi, 2018b). All students from this cohort gave informed consent to participate in the panel.

The dataset (SC5:10.0.0) contains 10 different waves of surveys (FDZ-LIfBi, 2018b) at different points in time. All analyses for this study come from wave 1 that was surveyed right after students’ university entrance.

### Sample and Sampling Procedures

The sampling applied students’ *study subject* as filter having STEM subjects as main focus. These were represented by a three-digit classification of the German Federal Statistical Office (Destatis [Statistisches Bundesamt], 2018a). Analyzing the proportion of females in each study subject, the results showed that the NEPS dataset had an oversampling, with 60% of all cases being female. Looking at the respective German data, the German Federal Statistical Office reports in its statistics on German university entrants in the winter term 2010/2011 (Destatis [Statistisches Bundesamt], 2018b) that only 50% were females. Therefore, each student was assigned a variable with the proportion of females within the first study subject based on the data provided by the German Federal Statistical Office (Destatis [Statistisches Bundesamt], 2018b). Students were classified with respect to their first study subject according to the number of females within it (see e.g., Ertl et al., 2014; low-proportion: less than 30% females; high proportion: more than 70% females; moderate proportion: percentages in between). The subjects were furthermore clustered according to their area of study (e.g., STEM, medicine, economics, educational sciences, and languages). This paper focuses on female students in STEM and distinguishes them according to subjects with a low proportion of females (less than 30%; STEM-L) and a moderate proportion of females (between 30 and 70%; STEM-M). The sample also includes medicine with a moderate proportion of females because this field is often discussed within the context of STEM careers (European Commission, 2015; Su and Rounds, 2015; Ertl et al., 2017).

The control sample for comparison included economics with a moderate proportion of females, educational sciences with a high proportion of females, and languages with a high proportion of females (mainly German, English, and the Romance languages). These sub-samples were selected because they had comparably high numbers of students in the respective category and no admission restrictions. With 12,936 out of a total sample of 17,910 students, the data set analyzed comprises more than 70% of the total data set.

### Variables and Analysis Procedures

The variables analyzed include:

• students’ RIASEC values,
• and their vocational aspirations as ISCO-08 codes.

Regarding the RIASEC values, we applied the NEPS-generated IILS-II scales values for each RIASEC dimension (see FDZ-LIfBi, 2018a, pp. 699–704). The IILS-II (interest inventory lifespan; Wohlkinger et al., 2011) is comprised of three items per dimension (two of them stemming from the AIST, Bergmann and Eder, 2005). Each had a range from one to five. The internal consistency of these scales was best for the social dimension and worst for the enterprising dimension (Cronbach’s a for Realistic: α = 0.704; Investigative: α = 0.625; Artistic: α = 0.629; Social: α = 0.749; Enterprising: α = 0.523; Conventional: α = 0.561). Please find the means and standard deviations for each dimension in Supplementary Table 1. Testing the hexagonal structure of vocational interests, we used Tracey’s (1997) program RANDALL to conduct the randomization test of hypothesized order relations (Hubert and Arabie, 1987). For our sample, the correspondence index (CI) was 0.81, *p* = 0.017, indicating a good fit of the data with the postulated hexagon. Students’ *three-letter codes* were generated from these values.

Students’ *vocational aspirations* were provided as ISCO-08 codes. The ISCO-08 codes were matched with the occupational information provided by O^∗^NET (2018) to obtain information about the RIASEC classification of these aspirations. This procedure had three steps: First, as there are more O^∗^NET-SOC codes than ISCO codes, RIASEC classifications of the O^∗^NET interest table were aggregated regarding the first six digits of the O^∗^NET-SOC code. This step focused on the O^∗^NET main categories that can be matched with ISCO codes. Second, the O^∗^NET-SOC codes were translated into ISCO codes based on the ISCO-08 to the 2010 SOC crosswalk table (Bureau of Labor Statistics, 2019). RIASEC values were aggregated in cases when the crosswalk provided more O^∗^NET codes as equivalent for one ISCO code. Finally, the resulting RIASEC classifications codes were assigned to the NEPS ISCO classifications of students’ vocational aspirations. In total, 9,860 out of 10,196 vocational aspirations (96.7%) were able to be RIASEC-classified via this procedure. Three hundred and eighteen vocational aspirations were NEPS coded as ISCO code 2100, which is a container category usually not foreseen as code in the ISCO-08 classification; the coding reflects a relatively vague aspiration in the context of doing something with technology. As this category is quite broad (mathematics and sciences), no RIASEC code could be assigned to those students’ aspirations, although they are in fact possibly part of the STEM fields. A total of 18 students (0.2%) without a RIASEC code for their vocational aspiration remained. Please find the means and standard deviations for the RIASEC dimensions of the aspirations in Supplementary Table 4.

All analyses were done with SPSS 25.0.

## Results

### Research Question 1: Students’ Interest Profiles

#### Students’ Interest Profiles

Table 1 gives an overview of the students’ interest profiles. The analysis shows that most females and males in all, but the STEM subjects had vector directions toward the same RIASEC dimensions in their interests. With STEM-M, 31.1% of the females had a predominant social dimension, while 19.8% of the males had a predominantly enterprising dimension. Differences also occurred for the STEM-L subjects in which 21.2% of the females had a predominant investigative dimension, while 34.5% of the males had a realistic dimension. With over 60%, educational sciences and females in the languages showed the most distinct vector directions, while overall the STEM subjects showed the lowest. This shows that students’ interests are more diverse for the STEM subjects than for other subject groups.

**Table 1:** Most- and second-frequent three-letter codes and vector directions derived from students’ interest values for the different subject areas including the proportion and absolute numbers (N) of students that showed the respective letter/codes.

	Female						Male					
	Three-letter code			Vector direction			Three-letter code			Vector direction		
	Code	Prop.	*N*	Dir.	Prop.	*N*	Code	Prop.	*N*	Dir.	Prop.	*N*
*STEM-L*	IRS	1.3	10	I	21.2	778	RIE	3.6	109	R	34.5	3041
	RIS	1.0	8				IRE	2.1	64			
*STEM-M*	ISE	2.7	49	S	31.1	1812	ISE	2.1	20	E	19.8	947
	SIE	2.3	42				IES	1.4	13			
*MED-M*	SIE	15.2	72	A	40.0	473	SEI	14.9	31	A	37.5	208
	SIA	4.4	21				SIE	9.6	20			
*ECO-M*	ECS	4.3	52	E	46.2	1196	ECS	4.4	34	E	53.9	775
	ESC	3.8	46				ESC	4.4	34			
*EDU-H*	SEA	8.6	89	S	68.1	1034	SEA	8.5	12	S	60.3	141
	SAE	7.0	73				SAE	6.3	9			
*Lang-H*	SEA	6.0	126	S	61.2	2100	SEA	4.2	17	S	49.0	406
	ASE	4.5	94				SEC	3.0	12			

*STEM-L, STEM subjects with a low proportion of females; STEM-M, STEM subjects with a medium proportion of women; MED-M, medicine (with a moderate proportion of females); ECO-M, economics (with a moderate proportion of females); EDU-H, education (with a high proportion of females); Lang-H, languages (with a high proportion of females). Code, frequent three-letter codes composed of the RIASEC dimensions: R, realistic; I, investigative; A, artistic; S, social; E, enterprising; C, conventional; Prop., proportion of three-letter codes or vector directions. Dir., frequent vector directions according to the RIASEC dimensions.*

We can recognize large variability when looking at the most frequent three-letter codes (Table 1). In almost all fields (except medicine) the occurrences of the most frequent code were below 10%, and even in medicine the most frequent code occurred just around 15% of the time. Particularly in the STEM fields we could see a high variability, with even the first most frequent codes contributing often less than 5%^2^; these codes were also comprised of opposite dimensions, e.g., R and S or I and E.

**FIGURE 3:**
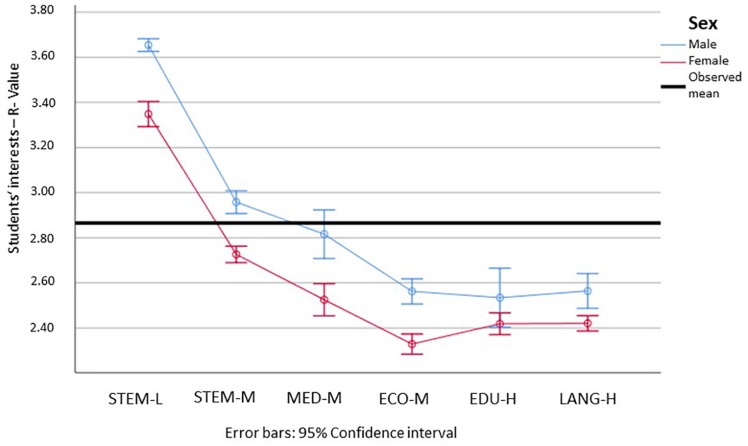
Means and 95% confidence intervals for the students’ realistic interests, for male (blue) and female (red) students in the different subject groups (min = 1; max = 5). STEM-L, STEM subjects with a low proportion of females; STEM-M, STEM subjects with a medium proportion of women; MED-M, medicine (with a moderate proportion of females); ECO-M, economics (with a moderate proportion of females); EDU-H, education (with a high proportion of females); Lang-H, languages (with a high proportion of females). Please find the means, standard errors, and confidence intervals for each group in Supplementary Table 2.

Looking at the things and people orientation, we can see significant differences in the realistic interests in all but the educational sciences (see Figure 3). Students’ realistic interests were noticeably higher in STEM-L than in all other fields. For females, the R values in STEM-M were higher than in the remaining other fields. For medicine, the means were between STEM-M and the remaining fields, although the confidence intervals were partially overlapping. Considering 3 as the scale mean, we can furthermore observe that students’ realistic interests in all but the STEM fields were below this.

**FIGURE 4:**
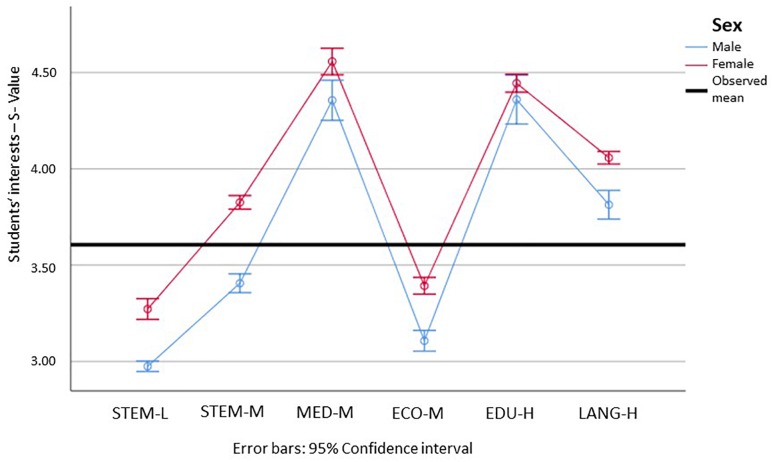
Means and 95% confidence intervals for the students’ social interests, for male (blue) and female (red) students in the different subject groups (min = 1; max = 5). STEM-L, STEM subjects with a low proportion of females; STEM-M, STEM subjects with a medium proportion of women; MED-M, medicine (with a moderate proportion of females); ECO-M, economics (with a moderate proportion of females); EDU-H, education (with a high proportion of females); Lang-H, languages (with a high proportion of females). Please find the means, standard errors, and confidence intervals for each group in Supplementary Table 3.

This is different for students’ social interests (see Figure 4). Here, we can see that all but the interests of the males in STEM-L were above the middle of the scale of 3. For the social interests, females showed significantly higher values for all but the educational sciences. Students showed the highest social interests in medicine and the educational sciences, and the lowest in STEM-L and economics, which showed significant but only marginally higher values than STEM-L. Social interests in STEM-M were significantly higher than in economics, and in the languages they were even higher than in STEM-M.

In light of the hypothesis that females have higher social interests (go more into people-related careers) and males have more realistic interests (go more into thing-related careers), we can verify this hypothesis for all but the educational sciences, which showed significant differences for neither of the dimensions. The results furthermore showed that STEM-L is the most things-oriented, and medicine and educational sciences are the most people-oriented. Economics is neither, and its values are among the lowest for both categories.

#### Interests Profiles of Students’ Vocational Aspirations

When it comes to students’ vocational aspirations, Table 2 provides insights into the three-letter codes and the main direction of the interest vectors. Considering the vector directions of students’ vocational aspiration, females and males had similar, and most frequent predominant letters in all areas but economics. Students’ aspirations in STEM-L had a focus on realistic activities. Students in STEM-M, educational sciences, and the languages had a focus on social activities. In medicine their focus was on investigative activities, while in economics the focus was on enterprising activities for the female students and on conventional ones for the males. These kinds results are also reflected in the distributions of the three-letter codes of the different aspirations.

**Table 2:** Most- and second-frequent three-letter codes and vector direction derived from students’ vocational aspirations for the different subject areas including the proportion and absolute numbers (*N*) of students that showed the respective letter/codes.

	Female						Male					
	Three-letter code			Vector direction			Three-letter code			Vector direction		
	Code	Prop.	*N*	Dir.	Prop.	*N*	Code	Prop.	*N*	Dir.	Prop.	*N*
*STEM-L*	RIC	9.4	73	R	52.8	472	IRA	10.5	320	R	65.5	1926
	IRA	6.7	52				IRC	9.4	288			
*STEM-M*	SAE	49.3	894	S	72.3	1533	SAE	42.3	401	S	60.1	735
	SAI/C	8.9	161				IRC	10.1	96			
*MED-M*	ISR	87.8	416	I	97.8	451	ISR	90.9	189	I	99.0	200
*ECO-M*	ECS	15.4	185	E	34.8	728	ECS	14.0	109	C	45.1	519
	SAE	6.3	76				CEI	11.0	86			
*EDU-H*	SIA	29.2	303	S	75.7	845	SIA	24.6	35	S	76.3	118
	SAE	13.3	138				SAE	15.5	22			
*Lang-H*	SAE	64.0	1346	S	90.5	1954	SAE	74.1	301	S	91.3	379
	SAI/C	13.2	277				AEC	3.9	16			

*STEM-L, STEM subjects with a low proportion of females; STEM-M, STEM subjects with a medium proportion of women; MED-M, medicine (with a moderate proportion of females); ECO-M, economics (with a moderate proportion of females); EDU-H, education (with a high proportion of females); Lang-H, languages (with a high proportion of females). Code, frequent three-letter codes composed of the RIASEC dimensions: R, realistic; I, investigative; A, artistic; S, social; E, enterprising; C, conventional; Prop., proportion of three-letter codes or vector directions. Dir., frequent vector directions according to the RIASEC dimensions.*

Focusing on the homogeneity of students’ vocational aspirations (Table 2), we can see extensive differences between the frequencies of students’ three-letter codes in STEM-L and STEM-M. While most students in STEM-M head into social professions with a high proportion of teaching activities, students in STEM-L show much more diverse aspirations.

**FIGURE 5:**
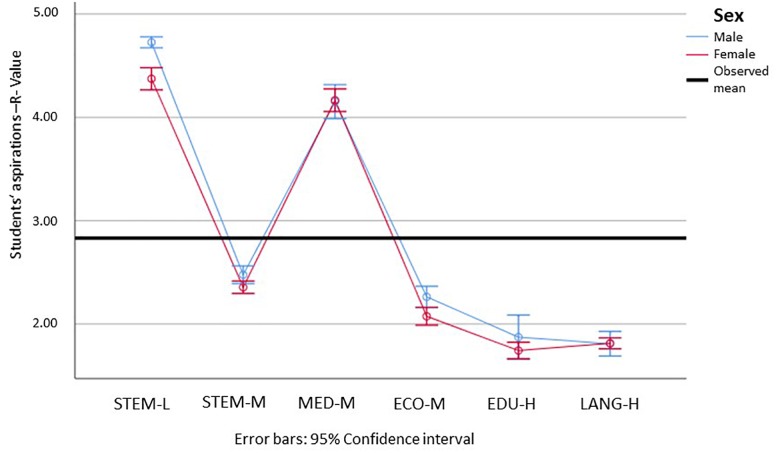
Means and 95% confidence intervals for interest profiles of students’ aspirations, realistic dimension, for male (blue) and female (red) students in the different subject groups (min = 1; max = 7). STEM-L, STEM subjects with a low proportion of females; STEM-M, STEM subjects with a medium proportion of women; MED-M, medicine (with a moderate proportion of females); ECO-M, economics (with a moderate proportion of females); EDU-H, education (with a high proportion of females); Lang-H, languages (with a high proportion of females). Please find the means, standard errors, and confidence intervals for each group in Supplementary Table 5.

**FIGURE 6:**
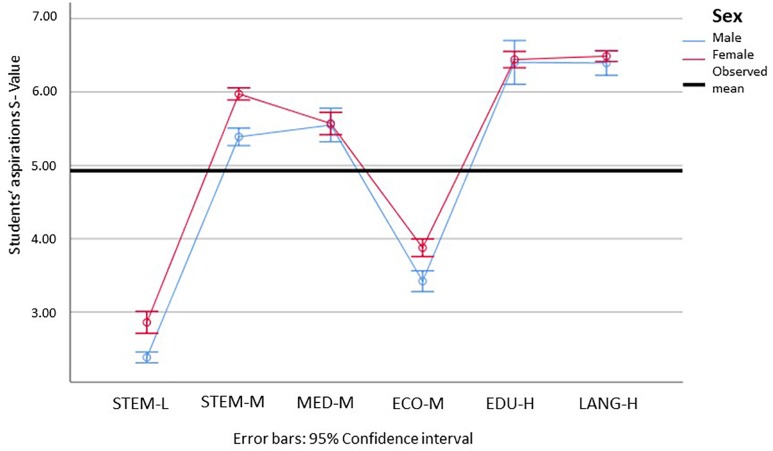
Means and 95% confidence intervals for interest profiles of students’ aspirations, social dimension, for male (blue) and female (red) students in the different subject groups (min = 1; max = 7). STEM-L, STEM subjects with a low proportion of females; STEM-M, STEM subjects with a medium proportion of women; MED-M, medicine (with a moderate proportion of females); ECO-M, economics (with a moderate proportion of females); EDU-H, education (with a high proportion of females); Lang-H, languages (with a high proportion of females). Please find the means, standard errors, and confidence intervals for each group in Supplementary Table 6.

Looking now at our hypothesis that males go more into things and females more into people, we can analyze the interest profiles of students’ vocational aspirations. In terms of the realistic or things dimension (see Figure 5), we can only observe one significant difference between females and males in STEM-L, which is noticeably small when comparing it with the differences between the STEM-L group and the other groups in the study. Of particular note is also that the medical aspirations have a very high amount of realistic interests, which is in contrast to the values regarding students’ individual realistic and social interests. When looking at the social interest profile of students’ aspirations (see Figure 6), we see several significant but small differences between males and females in STEM and economics. This indicates that females tend toward more social aspirations within each field of study. Comparing both dimensions, we can see that all, but the STEM-L and medical students’ aspirations have realistic values below the middle of the scale of 4. With the social dimension, almost all aspirations besides STEM-L and economics show high to very high characteristics of this dimension. This allowed us to partially verify the hypothesis that the fields of study are linked to differences in the things dimension and people dimension. However, for this particular student population, we obtained stronger evidence for the things dimension.

#### Interest Profiles of Students in STEM-L

**FIGURE 7:**
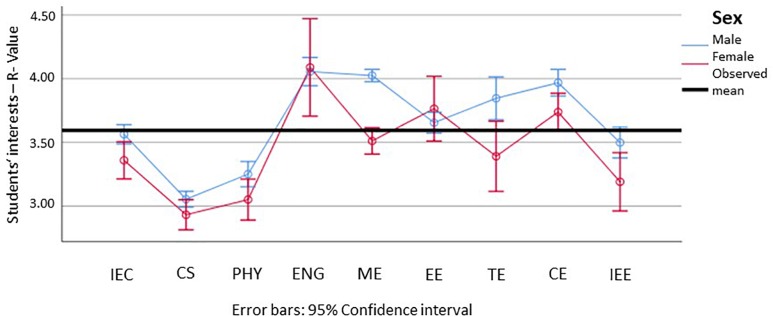
Means and 95% confidence intervals for the students’ realistic interests, for male (blue) and female (red) students in the different subject groups (min = 1; max = 5). IEE, Industrial Engineering, focus on economics; CS, Computer Science; PHY, Physics, Astronomy; ENG, Engineering, general; ME, Mechanical Engineering; EE, Electrical Engineering; TE, Traffic Engineering; CE, Civil Engineering; IEE, Industrial Engineering, focus on engineering. Please find the means, standard errors, and confidence intervals for each group in Supplementary Table 7.

Focusing now specifically on STEM-L, the following reports the data from subjects with more than 100 students in the sample. Regarding the realistic dimension, we can see that females score notably lower in all subjects except general and electrical engineering, and that the confidence intervals are generally larger for females than for males (which can be attributed to the reduced sample size). We can furthermore observe that the significant difference between males and females seen in Figure 3 disappears on the subject level for all subjects except mechanical engineering and traffic engineering (see Figure 7).

**FIGURE 8:**
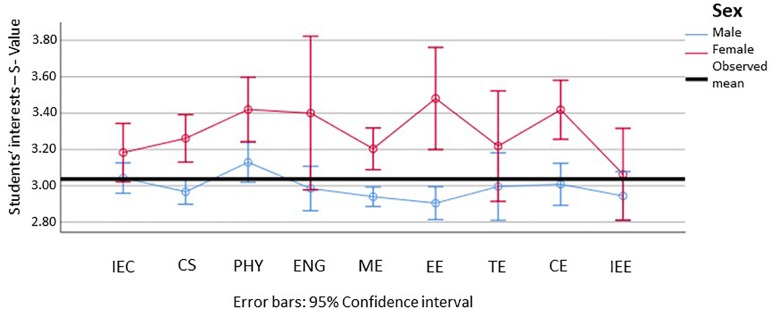
Means and 95% confidence intervals for the students’ social interests, for male (blue) and female (red) students in the different subject groups (min = 1; max = 5). IEE, Industrial Engineering, focus on economics; CS, Computer Science; PHY, Physics, Astronomy; ENG, Engineering, general; ME, Mechanical Engineering; EE, Electrical Engineering; TE, Traffic Engineering; CE, Civil Engineering; IEE, Industrial Engineering, focus on engineering. Please find the means, standard errors, and confidence intervals for each group in Supplementary Table 8.

Regarding the social dimension, we can generally observe higher values of females in this dimension, as well as generally higher confidence intervals of females (see Figure 8). Significant differences could be found for about half of the subjects including computer science, physics, and mechanical, electrical, and civil engineering.

Looking now at our hypothesis, we can still see significant differences persisting, although only for a few subjects. Of note here are the significant differences in the social dimension and partially in the realistic dimension.

### Research Question 2: Congruence Between Students’ Interests and Their Vocational Aspirations

Keeping in mind the significant gender differences regarding students’ individual interests and the comparably small differences in the field of occupational aspirations, it’s interesting to delve deeper into the congruencies of students’ interests and their aspirations. With the congruence vectors, it is important to note that a lower value means a higher congruence. The vector values are around 0.8 (see Table 3). Considering the maximum congruence of 0 and a theoretical minimum of 4, these vectors are within the highest quartiles for females as well for males, which indicates that students chose a subject that is in line with their individual interests. Descriptively, STEM subjects show lower congruencies, while educational sciences and languages show higher ones. The highest congruence is found with males in medicine, and the lowest with males in STEM-M.

**Table 3:** Mean length of students’ congruence vectors.

	Female			Male		
	*M*	*SD*	*N*	*M*	*SD*	*N*
*STEM-L*	0.963	0.341	470	0.941	0.320	1923
*STEM-M*	0.872	0.295	1533	0.973	0.304	735
*MED-M*	0.715	0.240	450	0.648	0.250	200
*ECO-M*	0.846	0.327	727	0.842	0.329	517
*EDU-H*	0.657	0.331	842	0.678	0.302	118
*Lang-H*	0.666	0.275	1952	0.777	0.296	379

*STEM-L, STEM subjects with a low proportion of females; STEM-M, STEM subjects with a medium proportion of women; MED-M, medicine (with a moderate proportion of females); ECO-M, economics (with a moderate proportion of females); EDU-H, education (with a high proportion of females); Lang-H, languages (with a high proportion of females).*

**FIGURE 9:**
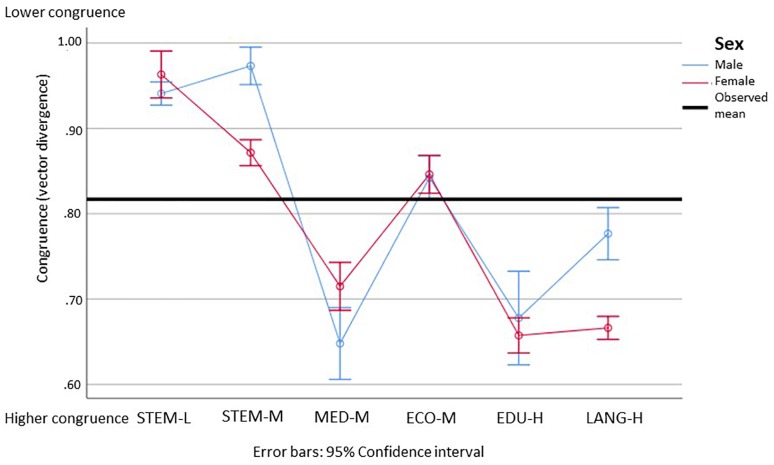
Means and 95% confidence intervals of students’ congruence between their interests and vocational aspirations for female (red) and male (blue) students based on an analysis with interest vectors (lower means indicate higher congruence). STEM-L, STEM subjects with a low proportion of females; STEM-M, STEM subjects with a medium proportion of women; MED-M, medicine (with a moderate proportion of females); ECO-M, economics (with a moderate proportion of females); EDU-H, education (with a high proportion of females); Lang-H, languages (with a high proportion of females). Please find the means, standard errors, and confidence intervals for each group in Supplementary Table 9.

Looking now at significant differences (Figure 9), gender differences are seen for STEM-M and the languages, with females showing a higher congruence in both areas. Regarding the differences between the subjects, females show the highest congruencies in educational sciences and the languages. These are slightly but significantly lower in medicine, lower again in STEM-M and economics, and the lowest in STEM-L. Males in contrast show the highest congruencies in medicine and educational sciences, lower congruencies in the languages, lower ones in economics, and the lowest in STEM.

**FIGURE 10:**
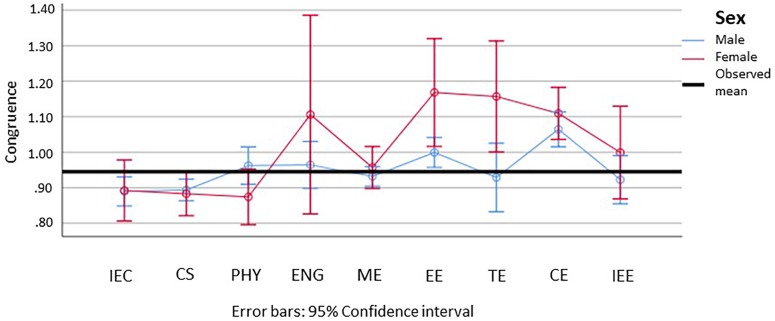
Means and 95% confidence intervals of students’ congruence between their interests and vocational aspirations for female (red) and male (blue) students based on an analysis with interest vectors (lower means indicate higher congruence). IEE, Industrial Engineering, focus on economics; CS, Computer Science; PHY, Physics, Astronomy; ENG, Engineering, general; ME, Mechanical Engineering; EE, Electrical Engineering; TE, Traffic Engineering; CE, Civil Engineering; IEE, Industrial Engineering, focus on engineering. Please find the means, standard errors, and confidence intervals for each group in Supplementary Table 10.

Looking more specifically at the STEM-L subjects, we were unable to identify significant differences for any of the subjects (see Figure 10). Descriptively, we can observe the high confidence intervals of females and a noticeably lower congruence, although this was not significant in electrical engineering and traffic engineering.

Looking at our alternative assumptions, we see that both are unable to satisfactorily explain the results. The assumption that students in fields in which they are under-represented also show a higher congruence regarding their interests and vocational aspirations could only be confirmed for male students in the educational sciences and the languages, and only when compared to males in STEM. Moreover, when interpreting the confidence intervals, it could be assumed that a higher sample size of females may in fact disclose that they show a significant lower congruence in STEM-L than males.

## Summary and Discussion

Summarizing our results, we can see on an *individual interest* level that females show significantly higher social interests, and males higher realistic ones across all study subjects except educational science. This expands the research of Su et al. (2009) by showing that the phenomenon of females having more social interests and males having more realistic ones holds across the disciplines. Of note, students in STEM-L showed a much higher level of realistic interests than all other fields, and a lower level of social interests than most other fields.

We obtained a quite different observation regarding students’ occupational aspirations. Here, we only found one significant difference between females and males in the realistic dimension in the area of STEM-L. This was also the case for the social dimension where we obtained less significant differences for the subjects of STEM and economics only. These differences were in line with the approach of Su and Rounds (2015) for predicting the proportion of females within a subject area via the difference in the things-people dimension. Interestingly, the significant differences held, consistent with Su and Rounds (2015), for the STEM-L area, which indicates that there are further factors affecting the proportion of females in this area. Overall, we observed the highest levels of realistic for occupational aspirations in STEM-L and medicine, and the lowest levels of social for occupational aspirations in STEM-L and economics.

Looking now at the congruencies between the individuals’ interests and the respective profile of their occupational aspiration, we could only see significant differences for STEM-M and the languages. In both, females showed a higher congruence than males. Remarkably, students in STEM and especially males in STEM-M showed the lowest levels of congruence, while students in education, males in medicine, and females in the languages showed the highest. Recalling our hypothesis (b) according to SCCT (Lent et al., 1994; Lent, 2013; Lent and Brown, 2013) that students show higher levels of congruence in fields where they are under-represented, we were unable to verify this, especially when it comes to females in STEM-L.

### Discussion

The paper started with the societal challenge to increase the number of STEM graduates (see Hingel et al., 2008). Looking, however, into students’ interest profiles and especially into the congruence between their interests and their vocational aspirations, we can see that this congruence is noticeably lower for STEM and especially for STEM-L than for any other of the fields analyzed – for females as well as for males. Keeping in mind that the analyses included 70% of a stratified student sample of German first year students, we have to assert that students’ interest profiles fit less for STEM environments than for other occupational environments.

#### The High Focus on Things as a Challenge for STEM Career Choices

One reason for this discrepancy may result from the strong focus on realistic interests, the *things* dimension, that STEM, especially STEM-L occupations require. We realized that students in STEM fields with a low proportion of females (STEM-L) showed noticeably higher realistic interests and lower social interests than male and female students in other subjects, including STEM fields with a moderate proportion of females (STEM-M). In other words, STEM fields in which students have high realistic interests and low social interests have a low proportion of females. Considering the strong gender differences regarding the things-orientation and people-orientation (Su et al., 2009; Morris, 2016), and in line with previous studies (Woodcock et al., 2013; Su and Rounds, 2015), we can state that men and women follow their gender-specific interests when choosing a study subject. In the current study, this was especially true for realistic interests because the subjects’ proportion of females was connected to the magnitude of the corresponding subject-specific mean of realistic interests.

This was less evident with respect to social interests, the *people* dimension, which were around or above the middle of the scale for all fields investigated. Besides STEM, also students of economics show a medium average in terms of social interests, which does not fit the moderate proportion of females. This would appear to be a combination of low realistic and low social interests that lead men and women to the decision to study economics. In addition, they on average show high enterprising and high conventional interests. Please find the means, standard errors, and confidence intervals for the enterprising and conventional dimension for each group in Supplementary Tables 11 and 12. Students in medicine with a moderate proportion of females on the other hand show social interests that are as high as or even higher than the mean social interests in study subjects with a high proportion of females (education, languages). This deviation is in line with the study of Su and Rounds (2015, p. 15) who expected a higher percentage of women in medical science given the observed gender differences in interests related to this field.

Similar differences between the study subjects can be observed regarding the things-orientation of students’ vocational aspirations. Here, students in STEM-L choose occupations that have a stronger realistic orientation than the occupations chosen by students in other fields of study. In addition, students in STEM-M aspire toward occupations with a higher realistic orientation than students in study subjects with a high proportion of women (education, languages). The only clear exceptions here are medical students aspiring toward medical occupations containing a relatively strong realistic orientation. This discrepancy is also in line with the study by Su and Rounds (2015, p. 15) who, based on the gender differences in interests, expected less than the actual proportion of women in the field of medical services. Su and Rounds (2015) conclude that there must be factors in addition to a high people-orientation (and a low things-orientation) within work environments that attract women (e.g., working conditions; e.g., Babarović et al., 2018). The magnitude of the mean social orientation of aspired occupations predominantly reflects the proportion of women in the different study subjects. Students in STEM-L aspire toward occupations with a low people-orientation, while students of STEM-M and medicine aspire toward occupations with a moderate people-orientation that is lower than the mean people-orientation in education and the languages. Again, the field of economics is characterized by a low people-orientation and a low things-orientation.

#### Effects of and Reasons for a Low Congruence Between Personal Interests and the Occupational Interest Profile

Compared to students in other subjects, students in STEM show smaller interest congruence with respect to their vocational aspirations. Apparently, students in STEM choose aspirations that are less compatible with their interest profiles. According to Holland (1997) and empirical evidence (e.g., Tsabari et al., 2005; Nye et al., 2012) lower congruence is connected to lower vocational satisfaction and performance. Thus, if students in STEM take the occupations they aspire it could be expected that they are less satisfied and perform lower than students of other fields.

In accordance to the RIASEC model people seek occupations that fit their interests and vocational environments seek people that fit their requirements. Since this tendency seems to be relatively weak in STEM, the question arises as to why a STEM subject or profession is chosen, even though one’s own interests do not fit (perfectly) with the requirements (or why a vocational environment chooses such an individual). As Gottfredson’s (1981, 2005) theory and the SCCT (Lent et al., 1994; Lent, 2013; Lent and Brown, 2013) point out there are multiple reasons for aspiring an occupation. Apart from fitting interests the sex-type or prestige of an occupation or outcome expectations like favorable prospects in the job market could be alternative reasons for an occupational choice. Vice versa, if there is a lack of candidates a vocational environment may also choose aspirants showing only moderate or weak fit.

Following the suggestions of Su and Rounds (2015), we aimed to investigate STEM fields on a finer level by distinguishing between different subjects within STEM-L. We assumed that within the different STEM-L subjects, the gender differences regarding realistic and social interests should vanish or at least decrease. This assumption could be confirmed for some but not all of the subjects, showing again that not all STEM fields are equal, even on a finer level.

### Limitations

The analyses rely on the huge sample of a large-scale panel study that applied professional sampling procedures. Here, the results are different from convenience samples applied in other studies of occupational interests. This strength, however, does in fact have some limitations, e.g., that only short scales of occupational interests could be processed. Although more detailed instruments would be desirable, the vector-analytical approach is very robust when it comes to analyzing congruencies in the context of these short scales. This robustness comes from the construction of the interest vectors as well as from the calculation of the Euclidean distance as a congruence measure. Both methods are far less sensitive to the side effects of short scales (e.g., equal values of dimensions) than ranking-based algorithms.

A further and more general problem when studying the characteristics of STEM fields on a fine level and in the context of other fields of study is the small number of women in work environments with a high things-orientation. The large confidence intervals in Figure 8 and 10 reflect this challenge. Considering that the NEPS SC5 has around 18,000 participants, it would be desirable to conduct an effective oversampling of under-represented student populations.

### For the Future

We could generally observe that students’ values for the realistic interests were comparably low, even for the STEM-L field, especially when compared to the social dimension. This may result from the construction of interest inventories for a wide population. Looking at the initial samples of interest inventories (e.g., Bergmann and Eder, 2005; or the meta-study of Su et al., 2009), it’s obvious that these were either mainly administered with school children or with a broad range of professions that may also include students or professions requiring a university degree. These inventories may, especially for the realistic dimension, comprise a high amount of skilled manual work activities that often don’t perfectly match occupations that students aspire to after finishing their university degrees. They hardly can account for different working profiles within occupations (this is by the way the pitfall of all kinds of interest inventories) even if O^∗^NET (2018) provides a very fine-grained structure of occupations. In any case, this will require further development in the interest inventories, e.g., by distinguishing the R dimension with respect to physical or manual technical work and more white-collar professions. Su et al. (2018) followed this approach by developing an eight-dimensional interest model.

## Conclusion

One of the most astonishing outcomes of this study is its low congruence of all students in STEM-L and the even lower congruence of males in STEM-M. This indicates a worse fit between individual interests and the vocational aspirations of students in these areas compared to students in other areas. At first glance, this finding appears similar to the results of Su and Rounds (2015) who had discrepancies in predicting the proportion of females in the areas with low proportions of females. However, it, has to be acknowledged that the approach of this study was different from Su and Rounds (2015). While they analyzed the things-people discrepancy on a macro level and compared this with the proportion of females within a subject area, our study compared the individual interests of students within different fields of study with the interest profile of the individual’s own occupational aspiration. Whatever the outcome, the results look similar: in the area of STEM-L either the proportion of females only vaguely meets the prediction or, in our case, in the area of STEM-L there are still remaining significant differences in both the realistic and social dimensions. This indicates that, especially for the area of STEM-L, other variables mediate the impact of interests. These may be, e.g., prestige (Gottfredson, 1981, 2005), stereotypes (Konrad et al., 2000; Ertl et al., 2014, 2017), aptitudes and motivational beliefs (Eccles and Wang, 2016), contextual variables like career-related network contacts (Lent, 2013; Lent and Brown, 2013), socialization factors (e.g., Bleeker and Jacobs, 2004), or working conditions (Ferriman et al., 2009; Moss-Racusin et al., 2012). These should be analyzed in a further study. For STEM-M on the other hand, we could observe that females show a noticeably higher congruence than males, although they are in fact the second-lowest for females in the subjects investigated. We also could observe a higher proportion of females aspiring to teaching or counseling professions in this field than males. Here, further research should investigate how far this difference results from females aspiring toward more social job profiles within an occupational area (see e.g., Gottfredson, 1981, 2005; Su and Rounds, 2015).

### Implications

The analyses in this paper show how necessary it is to distinguish different STEM subjects – at least with respect to the corresponding proportion of females. Just using the term “STEM” may blur obvious differences of subjects like biological science and engineering (see e.g., Ertl et al., 2014; Su and Rounds, 2015). Determining the proportion of females in a subject is one approach to distinguish this. It might also be worthwhile to develop a more fine-grained but researchable clustering of STEM in an effort to differentiate the investigated effects more effectively.

The theories above offer useful information in terms of bringing more women into STEM. According to Gottfredson (1981, 2005), possible steps to promote the realistic interests of girls/women should be taken at an early developmental stage before the things-orientated work environments are excluded due to a lack of consistency with the own self-concept. Consistent with the SCCT (Lent et al., 1994; Lent, 2013; Lent and Brown, 2013), interventions may also work at a later developmental stage provided that they are able to cause shifts in interests by influencing learning experiences, self-efficacy beliefs, and outcome expectations. We agree with Su and Rounds (2015) when they propose an emphasis on the social aspects of STEM fields. This approach may be more promising than the attempt to promote the development of a differentiated realistic interest profile and could generally accentuate the importance of social values when it comes to societal development.

## Ethics Statement

The analyses of this manuscript are secondary analyses of data published previously (Blossfeld et al., 2011). Data sources used for the analyses were the cohort of first year students(doi: 10.5157/NEPS:SC5:10.0.0) of the German National Educational Panel Study (Blossfeld et al., 2011). All students from this cohort gave informed consent to participate in the panel by providing their phone number for being contacted for telephone interviews after being informed about the purposes of the study. Specific information about the recruitment process can be found in the field report of the study (Steinwede and Aust, 2012). All data analyses were performed via a remote terminal (RemoteNEPS) at the LIfBi in Bamberg, Germany that provided a controlled privacy environment for data access. Furthermore, an ethics approval for the analyses was obtained by the local ethics committee.

## Author Contributions

All authors listed have made a substantial, direct and intellectual contribution to the work, and approved it for publication.

## Conflict of Interest Statement

The authors declare that the research was conducted in the absence of any commercial or financial relationships that could be construed as a potential conflict of interest.

## Footnotes

1 The method by Eder (1998) is similar to calculating the coordinates in the two-dimensional space using Prediger’s (1982) data/ideas and things/people dimensions. The two methods reveal identical results.

2 An equal distribution would show 0.83% for each code.
