# Supplementary material for: The Interest Profiles and Interest Congruence of Male and Female Students in STEM and Non-STEM Fields
Source: Front Psychol. 2019 Apr 30;10:897. doi: 10.3389/fpsyg.2019.00897 (PMC6502906; doi:10.3389/fpsyg.2019.00897)
Supplement: Supplementary file 1 [file Data_Sheet_1.pdf]

**Supplementary Materials**

Table SUP1: Means, standard deviations, and *Ns* for the RIASEC scales of students' occupational interests (Min. = 1; Max. = 5).

| | | Mean | *SD* | *N* |
|---|---|---|---|---|
| Realistic | male | 3.2409 | 0.95375 | 5519 |
| | female | 2.5847 | 0.82097 | 7393 |
| | total | 2.8651 | 0.93811 | 12912 |
| Investigative | male | 3.3006 | 0.86386 | 5519 |
| | female | 3.0186 | 0.94630 | 7393 |
| | total | 3.1391 | 0.92255 | 12912 |
| Artistic | male | 2.4158 | 0.85716 | 5519 |
| | female | 3.1581 | 0.97078 | 7393 |
| | total | 2.8408 | 0.99419 | 12912 |
| Social | male | 3.2164 | 0.89487 | 5519 |
| | female | 3.8966 | 0.83702 | 7393 |
| | total | 3.6059 | 0.92553 | 12912 |
| Enterprising | male | 3.3806 | 0.75266 | 5519 |
| | female | 3.4204 | 0.73283 | 7393 |
| | total | 3.4034 | 0.74161 | 12912 |
| Conventional | male | 2.6953 | 0.77319 | 5519 |
| | female | 2.8238 | 0.78701 | 7393 |
| | total | 2.7689 | 0.78369 | 12912 |

Table SUP2: Means, standard errors, and confidence intervals for Figure 3 – Students' realistic interests (Min. = 1; Max. = 5).

| | | | | 95%-Confidence Interval | |
|---|---|---|---|---|---|
| | | Mean | Standard Error | Lower Level | Upper Level |
| STEM-L | male | 3.655 | 0.014 | 3.627 | 3.683 |
| | female | 3.349 | 0.028 | 3.294 | 3.405 |
| STEM-M | male | 2.958 | 0.026 | 2.907 | 3.008 |
| | female | 2.726 | 0.019 | 2.689 | 2.762 |
| MED-M | male | 2.816 | 0.055 | 2.708 | 2.923 |
| | female | 2.526 | 0.036 | 2.455 | 2.597 |
| ECO-M | male | 2.561 | 0.028 | 2.506 | 2.617 |
| | female | 2.331 | 0.023 | 2.286 | 2.376 |
| EDU-H | male | 2.535 | 0.067 | 2.405 | 2.666 |
| | female | 2.419 | 0.025 | 2.371 | 2.467 |
| Lang-H | male | 2.564 | 0.039 | 2.487 | 2.641 |
| | female | 2.422 | 0.017 | 2.389 | 2.456 |

*Note.* STEM-L = STEM subjects with a low proportion of females; STEM-M = STEM subjects with a medium proportion of women; MED-M = medicine (with a moderate proportion of females); ECO-M = economics (with a moderate proportion of females); EDU-H = education (with a high proportion of females); Lang-H = languages (with a high proportion of females).

Table SUP3: Means, standard errors, and confidence intervals for Figure 4 – Students' social interests (Min. = 1; Max. = 5).

| | | Mean | Standard Error | 95%-Confidence Interval | |
|---|---|---|---|---|---|
| | | | | Lower Level | Upper Level |
| STEM-L | male | 2.975 | 0.014 | 2.948 | 3.003 |
| | female | 3.272 | 0.028 | 3.218 | 3.326 |
| STEM-M | male | 3.406 | 0.025 | 3.357 | 3.455 |
| | female | 3.826 | 0.018 | 3.790 | 3.861 |
| MED-M | male | 4.356 | 0.053 | 4.252 | 4.460 |
| | female | 4.557 | 0.035 | 4.488 | 4.627 |
| ECO-M | male | 3.108 | 0.028 | 3.054 | 3.162 |
| | female | 3.393 | 0.022 | 3.350 | 3.437 |
| EDU-H | male | 4.359 | 0.065 | 4.233 | 4.486 |
| | female | 4.444 | 0.024 | 4.397 | 4.491 |
| Lang-H | male | 3.814 | 0.038 | 3.739 | 3.888 |
| | female | 4.058 | 0.017 | 4.025 | 4.090 |

*Note.* STEM-L = STEM subjects with a low proportion of females; STEM-M = STEM subjects with a medium proportion of women; MED-M = medicine (with a moderate proportion of females); ECO-M = economics (with a moderate proportion of females); EDU-H = education (with a high proportion of females); Lang-H = languages (with a high proportion of females).

Table SUP4: Means, standard deviations, and *Ns* for the RIASEC scales of the interest profiles of students' occupational aspirations (Min. = 1; Max. = 7).

| | | Mean | *SD* | *N* |
|---|---|---|---|---|
| Realistic | male | 3.5682 | 1.88581 | 3877 |
| | female | 2.3533 | 1.31768 | 5983 |
| | total | 2.8310 | 1.67446 | 9860 |
| Investigative | male | 4.4759 | 1.89344 | 3877 |
| | female | 3.6487 | 1.61019 | 5983 |
| | total | 3.9740 | 1.77367 | 9860 |
| Artistic | male | 3.0785 | 1.38235 | 3877 |
| | female | 3.8271 | 1.27739 | 5983 |
| | total | 3.5328 | 1.36932 | 9860 |
| Social | male | 3.7695 | 2.37185 | 3877 |
| | female | 5.6769 | 1.95647 | 5983 |
| | total | 4.9269 | 2.32428 | 9860 |
| Enterprising | male | 3.7682 | 1.32062 | 3877 |
| | female | 3.8017 | 1.10274 | 5983 |
| | total | 3.7885 | 1.19321 | 9860 |
| Conventional | male | 3.8232 | 1.14660 | 3877 |
| | female | 3.3957 | 0.98727 | 5983 |
| | total | 3.5638 | 1.07326 | 9860 |

Table SUP5: Means, standard errors, and confidence intervals for Figure 5 – Students' occupational aspirations realistic profile (Min. = 1; Max. = 7).

| | | Mean | Standard Error | 95%-Confidence Interval | |
|---|---|---|---|---|---|
| | | | | Lower Level | Upper Level |
| STEM-L | male | 4.726 | 0.027 | 4.673 | 4.779 |
| | female | 4.373 | 0.055 | 4.266 | 4.480 |
| STEM-M | male | 2.477 | 0.044 | 2.391 | 2.562 |
| | female | 2.355 | 0.030 | 2.296 | 2.414 |
| MED-M | male | 4.152 | 0.084 | 3.987 | 4.316 |
| | female | 4.166 | 0.056 | 4.056 | 4.275 |
| ECO-M | male | 2.263 | 0.052 | 2.160 | 2.365 |
| | female | 2.075 | 0.044 | 1.988 | 2.161 |
| EDU-H | male | 1.873 | 0.109 | 1.658 | 2.087 |
| | female | 1.743 | 0.041 | 1.663 | 1.823 |
| Lang-H | male | 1.809 | 0.061 | 1.689 | 1.929 |
| | female | 1.813 | 0.027 | 1.761 | 1.866 |

*Note.* STEM-L = STEM subjects with a low proportion of females; STEM-M = STEM subjects with a medium proportion of women; MED-M = medicine (with a moderate proportion of females); ECO-M = economics (with a moderate proportion of females); EDU-H = education (with a high proportion of females); Lang-H = languages (with a high proportion of females).

Table SUP6: Means, standard errors, and confidence intervals for Figure 6 – Students' occupational aspirations social profile (Min. = 1; Max. = 7).

| | | Mean | Standard Error | 95%-Confidence Interval | |
|---|---|---|---|---|---|
| | | | | Lower Level | Upper Level |
| STEM-L | male | 2.383 | 0.038 | 2.309 | 2.457 |
| | female | 2.860 | 0.076 | 2.711 | 3.009 |
| STEM-M | male | 5.389 | 0.061 | 5.269 | 5.508 |
| | female | 5.974 | 0.042 | 5.891 | 6.057 |
| MED-M | male | 5.550 | 0.117 | 5.321 | 5.779 |
| | female | 5.570 | 0.078 | 5.417 | 5.723 |
| ECO-M | male | 3.421 | 0.073 | 3.279 | 3.563 |
| | female | 3.877 | 0.061 | 3.757 | 3.997 |
| EDU-H | male | 6.403 | 0.152 | 6.105 | 6.701 |
| | female | 6.442 | 0.057 | 6.330 | 6.553 |
| Lang-H | male | 6.394 | 0.085 | 6.228 | 6.561 |
| | female | 6.489 | 0.037 | 6.415 | 6.562 |

*Note.* STEM-L = STEM subjects with a low proportion of females; STEM-M = STEM subjects with a medium proportion of women; MED-M = medicine (with a moderate proportion of females); ECO-M = economics (with a moderate proportion of females); EDU-H = education (with a high proportion of females); Lang-H = languages (with a high proportion of females).

Table SUP7: Means, standard errors, and confidence intervals for Figure 7 – Students' realistic interests in STEM-L for the different subject groups: IEC = Industrial Engineering, focus on economics; CS = Computer Science; PHY = Physics, Astronomy; ENG = Engineering, general; ME = Mechanical Engineering; EE = Electrical Engineering; TE = Traffic Engineering; CE = Civil Engineering; IEE = Industrial Engineering, focus on engineering

| | | | | 95%-Confidence Interval | |
|---|---|---|---|---|---|
| | | Mean | Standard Error | Lower Level | Upper Level |
| IEC | male | 3.563 | 0.039 | 3.488 | 3.639 |
| | female | 3.359 | 0.074 | 3.214 | 3.504 |
| CS | male | 3.054 | 0.032 | 2.992 | 3.116 |
| | female | 2.932 | 0.060 | 2.814 | 3.050 |
| PHY | male | 3.251 | 0.051 | 3.151 | 3.350 |
| | female | 3.051 | 0.082 | 2.890 | 3.212 |
| ENG | male | 4.056 | 0.057 | 3.945 | 4.167 |
| | female | 4.089 | 0.195 | 3.706 | 4.472 |
| ME | male | 4.025 | 0.025 | 3.976 | 4.074 |
| | female | 3.511 | 0.053 | 3.408 | 3.615 |
| EE | male | 3.655 | 0.042 | 3.573 | 3.736 |
| | female | 3.765 | 0.130 | 3.510 | 4.019 |
| TE | male | 3.846 | 0.086 | 3.678 | 4.014 |
| | female | 3.391 | 0.140 | 3.115 | 3.666 |
| CE | male | 3.968 | 0.053 | 3.863 | 4.073 |
| | female | 3.739 | 0.075 | 3.592 | 3.885 |
| IEE | male | 3.499 | 0.062 | 3.377 | 3.620 |
| | female | 3.190 | 0.117 | 2.962 | 3.419 |

Table SUP8: Means, standard errors, and confidence intervals for Figure 8 – Students' social interests in STEM-L for the different subject groups: IEC = Industrial Engineering, focus on economics; CS = Computer Science; PHY = Physics, Astronomy; ENG = Engineering, general; ME = Mechanical Engineering; EE = Electrical Engineering; TE = Traffic Engineering; CE = Civil Engineering; IEE = Industrial Engineering, focus on engineering

| | | | | 95%-Confidence Interval | |
|---|---|---|---|---|---|
| | | Mean | Standard Error | Lower Level | Upper Level |
| IEC | male | 3.043 | 0.043 | 2.959 | 3.126 |
| | female | 3.183 | 0.082 | 3.022 | 3.343 |
| CS | male | 2.967 | 0.035 | 2.898 | 3.036 |
| | female | 3.261 | 0.067 | 3.130 | 3.392 |
| PHY | male | 3.129 | 0.056 | 3.019 | 3.239 |
| | female | 3.420 | 0.091 | 3.242 | 3.597 |
| ENG | male | 2.985 | 0.062 | 2.863 | 3.108 |
| | female | 3.400 | 0.216 | 2.977 | 3.823 |
| ME | male | 2.940 | 0.027 | 2.886 | 2.994 |
| | female | 3.204 | 0.059 | 3.089 | 3.319 |
| EE | male | 2.904 | 0.046 | 2.814 | 2.995 |
| | female | 3.480 | 0.143 | 3.200 | 3.761 |
| TE | male | 2.996 | 0.095 | 2.810 | 3.181 |
| | female | 3.218 | 0.155 | 2.914 | 3.523 |
| CE | male | 3.008 | 0.059 | 2.893 | 3.124 |
| | female | 3.418 | 0.083 | 3.256 | 3.580 |
| IEE | male | 2.944 | 0.068 | 2.810 | 3.078 |
| | female | 3.063 | 0.129 | 2.811 | 3.316 |

Table SUP9: Means, standard errors, and confidence intervals for Figure 9 – Congruence (lowest congruence = 4; highest congruence = 0).

| | | | | 95%-Confidence Interval | |
|---|---|---|---|---|---|
| | | Mean | Standard Error | Lower Level | Upper Level |
| STEM-L | male | 0.941 | 0.007 | 0.927 | 0.954 |
| | female | 0.963 | 0.014 | 0.936 | 0.991 |
| STEM-M | male | 0.973 | 0.011 | 0.951 | 0.995 |
| | female | 0.872 | 0.008 | 0.856 | 0.887 |
| MED-M | male | 0.648 | 0.021 | 0.606 | 0.690 |
| | female | 0.715 | 0.014 | 0.687 | 0.743 |
| ECO-M | male | 0.842 | 0.013 | 0.816 | 0.868 |
| | female | 0.846 | 0.011 | 0.824 | 0.868 |
| EDU-H | male | 0.678 | 0.028 | 0.623 | 0.733 |
| | female | 0.657 | 0.010 | 0.637 | 0.678 |
| Lang-H | male | 0.777 | 0.016 | 0.746 | 0.807 |
| | female | 0.666 | 0.007 | 0.653 | 0.680 |

*Note.* STEM-L = STEM subjects with a low proportion of females; STEM-M = STEM subjects with a medium proportion of women; MED-M = medicine (with a moderate proportion of females); ECO-M = economics (with a moderate proportion of females); EDU-H = education (with a high proportion of females); Lang-H = languages (with a high proportion of females).

Table SUP10: Means, standard errors, and confidence intervals for Figure 10 – Congruence in STEM-L for the different subject groups: IEC = Industrial Engineering, focus on economics; CS = Computer Science; PHY = Physics, Astronomy; ENG = Engineering, general; ME = Mechanical Engineering; EE = Electrical Engineering; TE = Traffic Engineering; CE = Civil Engineering; IEE = Industrial Engineering, focus on engineering

| | | | | 95%-Confidence Interval | |
|---|---|---|---|---|---|
| | | Mean | Standard Error | Lower Level | Upper Level |
| IEC | male | 0.890 | 0.021 | 0.849 | 0.930 |
| | female | 0.892 | 0.044 | 0.806 | 0.978 |
| CS | male | 0.894 | 0.016 | 0.863 | 0.924 |
| | female | 0.883 | 0.031 | 0.821 | 0.945 |
| PHY | male | 0.962 | 0.027 | 0.910 | 1.015 |
| | female | 0.874 | 0.040 | 0.796 | 0.952 |
| ENG | male | 0.964 | 0.034 | 0.898 | 1.030 |
| | female | 1.106 | 0.143 | 0.826 | 1.386 |
| ME | male | 0.932 | 0.014 | 0.904 | 0.959 |
| | female | 0.957 | 0.030 | 0.898 | 1.016 |
| EE | male | 0.999 | 0.022 | 0.957 | 1.042 |
| | female | 1.168 | 0.077 | 1.016 | 1.320 |
| TE | male | 0.929 | 0.049 | 0.832 | 1.025 |
| | female | 1.157 | 0.080 | 1.001 | 1.313 |
| CE | male | 1.065 | 0.025 | 1.015 | 1.114 |
| | female | 1.109 | 0.037 | 1.036 | 1.183 |
| IEE | male | 0.923 | 0.035 | 0.855 | 0.991 |
| | female | 0.999 | 0.067 | 0.869 | 1.130 |

Table SUP11: Means, standard errors, and confidence intervals for students' enterprising interests (Min. = 1; Max. = 5).

| | | Mean | Standard Error | 95%-Confidence Interval | |
|---|---|---|---|---|---|
| | | | | Lower Level | Upper Level |
| STEM-L | male | 3.252 | 0.013 | 3.227 | 3.278 |
| | female | 3.145 | 0.026 | 3.094 | 3.196 |
| STEM-M | male | 3.346 | 0.023 | 3.300 | 3.392 |
| | female | 3.277 | 0.017 | 3.243 | 3.310 |
| MED-M | male | 3.381 | 0.050 | 3.283 | 3.479 |
| | female | 3.306 | 0.033 | 3.241 | 3.371 |
| ECO-M | male | 3.787 | 0.026 | 3.737 | 3.838 |
| | female | 3.632 | 0.021 | 3.591 | 3.673 |
| EDU-H | male | 3.592 | 0.061 | 3.473 | 3.710 |
| | female | 3.477 | 0.022 | 3.433 | 3.521 |
| Lang-H | male | 3.578 | 0.036 | 3.508 | 3.648 |
| | female | 3.525 | 0.016 | 3.494 | 3.556 |

*Note.* STEM-L = STEM subjects with a low proportion of females; STEM-M = STEM subjects with a medium proportion of women; MED-M = medicine (with a moderate proportion of females); ECO-M = economics (with a moderate proportion of females); EDU-H = education (with a high proportion of females); Lang-H = languages (with a high proportion of females).

Table SUP12: Means, standard errors, and confidence intervals for students' conventional interests (Min. = 1; Max. = 5).

| | | Mean | Standard Error | 95%-Confidence Interval | |
|---|---|---|---|---|---|
| | | | | Lower Level | Upper Level |
| STEM-L | male | 2.652 | 0.014 | 2.625 | 2.680 |
| | female | 2.866 | 0.028 | 2.812 | 2.921 |
| STEM-M | male | 2.797 | 0.025 | 2.748 | 2.846 |
| | female | 2.894 | 0.018 | 2.858 | 2.929 |
| MED-M | male | 2.322 | 0.053 | 2.217 | 2.427 |
| | female | 2.478 | 0.035 | 2.408 | 2.547 |
| ECO-M | male | 2.897 | 0.028 | 2.843 | 2.951 |
| | female | 3.027 | 0.022 | 2.984 | 3.071 |
| EDU-H | male | 2.498 | 0.065 | 2.371 | 2.624 |
| | female | 2.637 | 0.024 | 2.590 | 2.684 |
| Lang-H | male | 2.654 | 0.038 | 2.579 | 2.728 |
| | female | 2.803 | 0.017 | 2.770 | 2.835 |
